# Design Service Life of RC Structures with Self-Healing Behaviour to Increase Infrastructure Carbon Savings

**DOI:** 10.3390/ma14123154

**Published:** 2021-06-08

**Authors:** Ana Bras, John Milan van der Bergh, Hazha Mohammed, Ismini Nakouti

**Affiliations:** 1 Built Environment and Sustainable Technologies (BEST) Research Institute, Liverpool John Moores University, Liverpool L3 2ET, UK; J.M.VanDerBergh@2019.ljmu.ac.uk (J.M.v.d.B.); H.B.Mohammed@2018.ljmu.ac.uk (H.M.); 2 Centre for Natural Products Discovery (CNPD), School of Pharmacy and Biomolecular Sciences, Liverpool John Moores University, Liverpool L3 3AF, UK; I.Nakouti@ljmu.ac.uk

**Keywords:** self-healing, concrete, durability, low carbon impact, sustainability

## Abstract

Corrosion of reinforced concrete (RC) structures costs the UK GBP 23b annually and is one of the main durability problems contributing to the development of rust, spalling, cracking, delamination, and structural deterioration. This paper intends to demonstrate the benefit of using tailored self-healing bacteria-based concrete for RC corrosion minimisation and service life increase. The purpose was to evaluate the enhancement in the lifespan of the structure exposed to a harsh marine microenvironment by utilising a probabilistic performance-based method. Comparison is made with the performance of a commercially available solution and in terms of embodied carbon impact. Three different concretes, using CEM I 52.5N, CEM II/A-D, and CEM III/A, were tested with and without an iron-respiring bioproduct (BIO) and an added admixture corrosion inhibitor (AACI). Results show that bioproduct significantly contributes to service life increase of RC structures with CEMIII/A. The repair solution with self-healing behaviour not only increases RC service life, but also enables us to decrease the required cover thickness from 60 mm to 50 mm in an XS2 chloride environment. In both XS2 and XS3 environments, a comparison of CEMIII/A+BIO and CEMII/A-D+AACI concrete shows the benefit of using bioproduct in corrosion inhibition context, besides contributing to an embodied carbon reduction of more than 20%.

## 1. Introduction

### 1.1. General Context

The durability of reinforced concrete (RC) structures in terms of corrosion is now of significant concern. The maintenance and repair cost of the UK’s existing structures is approximately ~GBP 40 billion/year, with the majority of structures being made using RC. High labour costs related to infrastructure maintenance are incurred; on the other hand, damage to nature may typically be self-healed, and the durability of structures increased; therefore, learning from nature is crucial. Such impacts on infrastructure occur globally, for instance, in India, where RC corrosion costs between 3–4% of the annual national gross domestic product (with an upward trend due to increased construction levels and lack of regulation).

There is currently a significant gap between predicted and real behaviour of structures, as real behaviour is not yet well understood [1,2]. As a consequence, the actual CO2 emissions can be double those anticipated at the design stage due to: uncertainty in material properties or design parameters; incorrect assumptions of occupant and building behaviour; influence of climatic conditions, etc. [3,4,5]. To design and build more efficiently is a necessary prerequisite to increase structures’ resilience, reduce the carbon dioxide emissions by half, and minimise the performance gap that is often found from a structural and energy perspective. The design of minimum material instead of minimum cost could significantly reduce the construction materials used in buildings and structures, which would result in an analogous decrease in embodied carbon emissions. This would contribute to the minimisation of the gap between the designed and real behaviour of a structure or building. Closing the performance gap in the built environment would facilitate dramatic reductions in operational and embodied energy use.

The concepts of durability design of RC structures [6,7,8,9] and the same prescriptive approaches in conjunction with conventional repair techniques have been proposed for decades. The current state-of-the-art additionally involves approaches that can be either complementary or alternative to conventional repair, including different solutions, from repair principles to defensive electrochemical processes, inhibitors of chemical corrosion, or surface protective coatings [10]. The existing design rules are, in general, presented as deem-to-satisfy rules; however, they do not give direct insight into the service life, the necessary maintenance, or, e.g., the probability of premature failure, which increases the gap between predicted and real behaviour; therefore, waste of construction materials, higher CO2 emissions, higher maintenance costs, and lack of comfort in buildings are some of the major consequences.

This economic and environmental burden can be reduced through better evaluation of RC lifecycles and by using durable materials. Performance parameters allow the desired functions of the constructive solutions to be identified and clearly defined, contributing to the optimisation of the quantity of materials needed and reducing carbon consumption. Performance-based approaches are the future of standards; however, to support the designer in the decision-making process, a great deal of knowledge is required.

**Figure 1:**
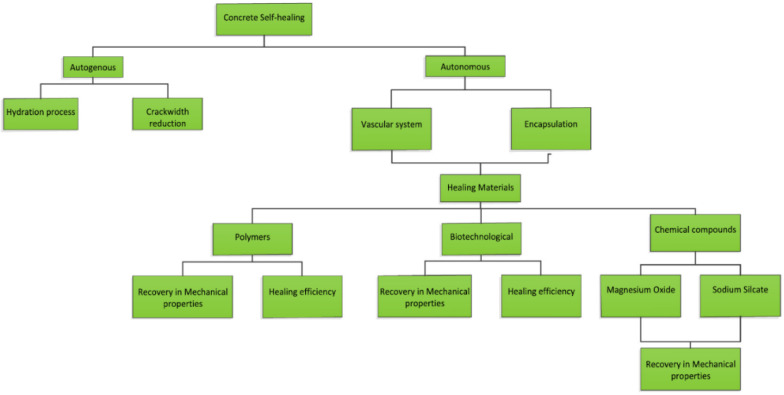
Different types of self-healing in concrete [12].

Structures mimicking nature and self-healing are of great interest, with the durability assessment of concrete being crucial for understanding the expected service life. Self-healing materials have the capability to repair themselves back to their original state [11]. In the last decades, there has been significant research interest in self-healing concrete, with various solutions being proposed and produced. Concrete self-healing can be divided into two main types: autonomous and autogenous (Figure 1). Autonomous self-healing requires intended additives to be combined for healing purposes in a cementitious matrix and also utilises specific techniques for transporting these additives, while autogenous healing is a natural crack repair mechanism that may take place in concrete. However, there is a lack of systematic evaluation and comparison methods of the healing efficiency process in construction materials, which limits possible application in practice. The current methods mainly work by healing some damage that occurred in the material, and only a few of them focused on the deterioration of concrete due to a reduction of pH value or ion intrusion. The RC structure’s durability with respect to corrosion is of considerable concern at present, leading to a major impact on the life cycle sustainability of structures and buildings. The use of performance-based design to minimise RC corrosion combined with self-healing methods to improve materials performance is a novelty that deserves to be explored.

Corrosion of steel is the primary cause of these structures’ degradation and is caused by the environmental conditions around them. Apart from $O_{2}$ and $H_{2}O$, which are necessary components for the corrosion reactions, chloride ions (${\mathrm{Cl}}^{-}$) and carbon dioxide (${\mathrm{CO}}_{2}$) are the two environmental factors that play a major role in corrosion processes. Efficient self-healing material must seal the cracked walls in order to minimise permeability, have potential activity in the long-term, be consistent with the matrix of concrete, support multiple event processing, and be economically viable.

For an autonomous self-healing function, materials could be categorised as bacterial, polymers, and chemical compounds (magnesium, specifically and sodium silicate) [12]. Little consideration is usually given to the long-term durability of the concrete with different binders, with and without self-healing behaviour, taking into account their applicability for commercial-scale use.

### 1.2. Effect on Corrosion Inhibition of RC

#### 1.2.1. Commercially Available Solutions

Alternative or supplementary techniques to traditional repair of RC are well known [7,8,13,14,15]. Corrosion inhibitors are a possible solution for repair. As a function of the specific action, there are three different forms of inhibitors: anodic, cathodic, and mixed inhibitors [16]. Based on that, three main examples are presented:

(a) Anode-acting inhibitors: sodium nitrate (inorganic compound) and calcium nitrite.
(b) Oxygen reaction inhibitors surrounding cathodes: sodium carbonate and sodium hydroxide (inorganic compounds).
(c) Anodic and cathodic region-acting inhibitors: amino alcohols and amino carboxylates (organic compounds).

Inhibitors of corrosion for repairing RC structures could be used as a sole measure or combined together with conventional repair methods, enabling a multi-barrier approach; therefore, they may be incorporated as a fresh concrete admixture (AACI) or applied on the surfaces of repaired hardened concrete, acting as an inhibitor of migrating corrosion. Their effect relies on acting mainly on the initiation period of corrosion and less on the propagation part [17,18].

#### 1.2.2. Bacterial Bio-Healing Solutions

(a) Precipitation of calcium carbonate for self-healing of concrete impact on corrosion

Microbiological precipitation of calcium carbonate (MICP) is a biotechnological healing technique that utilises the ability of certain types of bacteria to induce the formation of calcium carbonate inside the cementitious matrix. One of the most extensively studied and used bacterial genera for this purpose is the *Bacillus* genus, which contains species that are highly resistant to the alkaline environment [19] and are sporulating, which means they are resistant to unfavourable conditions (low/high temperatures, lack of moisture etc.). Based on [20,21], an approach for bacterial healing consists of the following:

1. To provide a source of bacterial nutrients.
2. The production of calcium carbonate as a consequence of the organic processes, such as: urea hydrolysis production, sulphate reduction, and photosynthesis.

The working hypothesis is that these bacteria are able to produce the precipitations of calcium carbonate and of biologically inducing chemical precipitations in which the organism develops an ideal mineral phase extracellular micro-environment, identified as bio-mineralization. Sometimes, the metabolic pathway for calcium carbonate generation can have an impact on the corrosion of cement-based materials [22,23,24,25,26,27,28,29].

The existing state-of-the-art emphasises that the production of ammonium through the ureolytic pathway could cause the production of ammonium salts, which could lead to corrosion or conversion of ammonium to nitric acid, which might damage the structure of the concrete [23]. Aerobic heterotrophic conversion of organic compounds needs a high degree of calcium source for the development of calcite when compared to the ureolytic pathway [30]. High levels of calcium salts, however, can pose a danger to concrete structures as well. The ureolytic pathway has greater effectiveness than denitrification in soil improvement applications, as the enzymatic urea hydrolysis occurs in contrast to other pathways in a much shorter time span; therefore, it is recognised as the quickest path to calcium carbonate development and healing the cracks [23], despite the fact that the denitrification pathway produces twice as much of carbonate per mole of electron than ureolytic [29]. The use of these bacteria-based solutions is still currently limited [24,31,32,33] not only for the reasons mentioned above, but also because there is a lack of systematic characterisation of properties associated with the corrosion process in reinforced concrete structures.

(b) Microbial iron respiration as a contribution to corrosion inhibition in self-healing concrete

Although certain bacterial strains are known to be able to induce or promote the corrosion of steel (e.g., anaerobic sulphate-reducing bacteria), other strains can inhibit corrosion either by directly depleting the $O_{2}$ necessary for the oxidation of iron, or indirectly by using Fe(II) to reduce $O_{2}$ [34]. It is crucial to understand how biofilm grows in RC as it affects the chemistry of the environment around the steel at different stages of biofilm development, creating an impact on service life. Another biotechnological approach to prolonging the service life of RC, specifically the rebars themselves, is using iron-respiring bacteria.

The most common form of corrosion is rusting in the presence of water and oxygen, which can be described using well-known chemical reactions, starting with the reduction of metallic iron to Fe(II)—Equation (1):

(1) $$2\mathrm{Fe}+{\;O}_{2}+2H_{2}O\overset{}{\to }2{\mathrm{Fe}}^{2+}+4{\mathrm{OH}}^{-}$$

Fe(II) is further reduced to Fe(III)—Equation (2)—which then forms amorphous iron(III)-hydroxide—Equation (3):

(2) $$4{\mathrm{Fe}}^{2+}+O_{2}+2H_{2}O\overset{}{\to }4{\mathrm{Fe}}^{3+}+4{\mathrm{OH}}^{-}$$

(3) $${\mathrm{Fe}}^{3+}+3{\mathrm{OH}}^{-}\overset{}{\to }\mathrm{Fe}{\left(\mathrm{OH}\right)}_{3}$$

Finally, after dehydration, $\mathrm{Fe}{\left(\mathrm{OH}\right)}_{3}$ decomposes to porous ${\mathrm{Fe}}_{2}O_{3}$ and water. Thus, by eliminating the supply of $O_{2}$, iron-respiring bacteria impede steel corrosion. In addition to the aforementioned oxygen consumption by respiring aerobic microorganisms inside the biofilm, resulting in a diminution in the reactant on the surface of the metal, there are several mechanisms most commonly cited for the inhibition. These are: (1) the formation of a diffusion barrier to corrosion products that stifles metal dissolution, (2) the production of metabolic products acting as corrosion inhibitors (e.g., siderophores or specific antibiotics in order to prevent the proliferation of corrosion-causing organisms such as sulphate-reducing bacteria), and (3) the passive layer formation, which is unique to the presence of microorganisms [35].

The Fe(III)-respiring bacteria usually couple the oxidation of the organic substrate, such as acetate or lactate, to reduce Fe(III) to Fe(II). In a study performed by Dubiel et al. [36], electrochemical impedance spectroscopy has been used in order to assess the rate of the corrosion and the corrosion potential as a function of the time for these mutants, and biofilms consisting of iron-respiring bacteria have been shown to inhibit steel corrosion rate rather than accelerating it. The inhibition of corrosion tends to be due to the reduction of ferric ions to ferrous ions and increased oxygen intake, all of which are direct implications of microbial respiration.

This research work intends to demonstrate that the most effective repair method for RC structures should be regarded as a long-term environmental impact feature. Particularly, this should take into account the embodied carbon expenditure and the longevity of the maintenance. The purpose was to evaluate the enhancement in the lifespan of a structure exposed to a harsh marine microenvironment by utilising a probabilistic performance-based method. The best alternative repair method for RC structures was selected by considering 100 years of service life and a maximum appropriate probability of achieving steel reinforcement corrosion of 2.3%, in compliance with Eurocode 0 requirements for the durability class RC3. Performance-based design using a probabilistic approach was utilised to compare the long-term behaviour of three different concrete compositions repaired with an iron-respiring bioproduct and a chemical-corrosion inhibitor.

## 2. Materials and Methods

### 2.1. Materials

**Figure 2:**
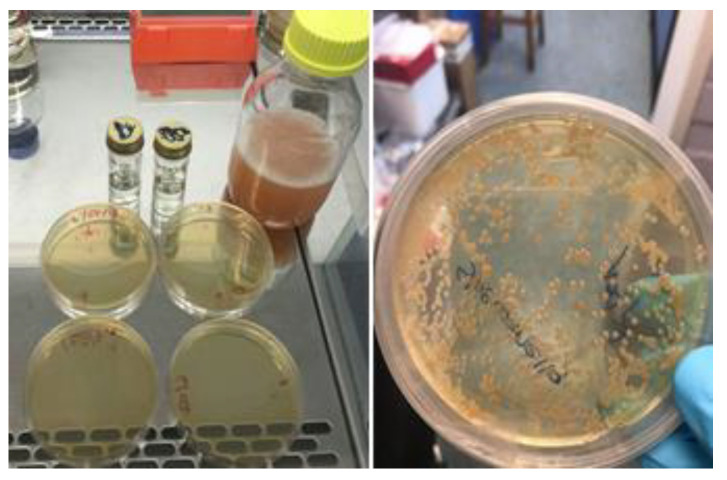
Serial dilution of the bio-agent.

In the laboratory, three distinct compositions of concrete were manufactured in order to simulate the actual characteristics of concrete that can be used in the RC structures repair situation (Table 1), such as in bridges exposed to maritime environments: CEM I 52.5 N, CEM II/A-D, and CEM III/A concrete types with and without an iron-respiring bioproduct. The performance of the bioproduct was later compared to an added admixture corrosion inhibitor (AACI). The AACI in question was MCI 2005^®^ (from Cortec^®^ Corporation, St. Paul, MN, USA) with a concentration of 0.60 L per m^3^ of concrete. The performance results of the AACI are included in the Andrade Report [17,37]. The iron-respiring bioproduct contained a bacterial *Shewanella*-like strain (iron-respiring bacteria), Figure 2, with a ratio of 2.1%/binder. The durability properties and the compressive strength of the three mixes of concrete with and without bioproduct or AACI were studied as it relates to water absorption via capillary, a rapid non-steady state chloride test, and the surface electrical resistivity.

Three samples per composition with 150 mm size concrete cubes were loaded to failure according to EN 12390-4 [38]. Capillary tests were carried out according to EN 1015-18 [39]. For a period of 8 days, the samples were put in a drying cabinet at 30 °C until the obtained change of mass was under 0.1%. The samples mass and water absorption were recorded at 0′, 5′, 15′, 30′, 1 h, 2 h, 3 h, 21 h, up to 28 days, until the absorption of water reached the asymptotic value. The chloride migration coefficient for each concrete composition was determined by the NT BUILD 492 method and represents a measure of the resistance of the tested material to chloride penetration. According to AASHTOTP 95 [40] and ASTM C1202 [41], electrical resistivity has been achieved and is well associated with certain concrete performance characteristics, such a water absorption, embedded steel corrosion rate, and chloride diffusion coefficient.

Table 1 shows the dosage of the cement content and the water/cement (w/c) ratio in the concretes for a compressive strength target of C40/50, suitable for columns in bridges. The concrete composition mixtures were designed in compliance with EN 197-1 (2000) [42] and EN 206-1 [43], and six samples were produced for each concrete composition.

For each concrete composition, three scenarios were then defined:

1. Control—plain concrete;
2. Concrete containing admixture inhibitor;
3. Concrete containing bioproduct for the purpose of self-healing behaviour.

### 2.2. Probabilistic Analysis of RC Service Life

A probabilistic approach was used for the design lifetime calculations, where assumptions, parameters, and mathematical models for the lifetime computation are based on fib Bulletin 34 (2006) [8], EN 206-1 [43], the LNEC E465 (2009) [44] specification, and [45]. The Monte Carlo method is used for the probabilistic analysis of lifetime design. The RC structure design service life (*t_L_*), when exposed to corrosion, is the sum of the initiation period (*t_i_*) and propagation period (*t_p_*), where the *t_L_* must present higher values than the predicted working life (*t_g_*) that was decided to adopt for a structure. The performance is a function of the actions (*S*), such as Cl^−^ migration, and resistance (*R*), such as the cover of concrete and the concrete characteristics. The risk measure associated with the particular event of *R*(*t*) < *S*(*t*) could be defined as the likelihood of failure [17,18]. This means that the probability of failure corresponds to the probability that the limit state function (*t_L_-t_g_*) is negative.

The initiation period resulting from the actions of chlorides follows the recommendations of fib Bulletin 34 [8], adopting here the Fick’s 2nd law (that takes into account the time effect in the diffusion process). The initiation period is expressed by Equation (4).

(4) $$t_{i}={\left[{\left(\frac{2}{c}\;erf^{-1}\left(1-\frac{C_{R}-C_{i}}{C_{S}-C_{i}}\right)\right)}^{-2}\frac{1}{\;k\;D_{0}\;t_{0}{}^{n}}\right]}^{\frac{1}{1-n}}$$

where *c* is the cover of concrete, *erf* is the function of error, *C_R_* is the amount of chloride threshold by weight of the cement (%), *C_S_* is the amount of surface chloride by weight of cement (%), *C_i_* the initial amount of chloride within the concrete mass by weight of cement (%), *k* is the product of parameters that considers curing (*K_D,C_*), temperature (*K_D,T_*), relative humidity (*K_D,RH_*), and *n* is the factor of ageing. These values are a function of the exposure class (XS2 or XS3) and are available at the Technical Specification LNEC E 465 from EN 206-1 regarding the methodology for estimating the service life of RC structures.

Equation (5) presents the variables and equation for estimating the propagation period in the chloride environments. Faraday’s law was used to calculate the propagation period, where the radius reduction factor of reinforcement the steel was attained from the empirical equation (Equation (6)).

(5) $$t_{p}=y\;\varphi_{0}\;\frac{1}{1.15\;\alpha \;i_{corr}}$$

where *t_p_* (years) is the period of propagation, *φ_0_* (mm) is the original steel reinforcement diameter, and *φ_0_* = 10 (pitting corrosion, as this is more common in chloride environments, which is more concentrated and intensive at various steel cross-section spots). The factor of radius reduction *y* (mm) is presented as:

(6) $$y=\left(74.5+7.3\;\frac{c}{\varphi_{0}}-17.4\;f_{\mathrm{td}}\right)\;\frac{0.2}{\varphi_{0}}$$

where f_td_ is the tensile strength of the concrete cover.

According to the experimental results obtained in the Andrade Report [37] on the added admixture corrosion inhibitor (MCI 2020 ^®^, Cortec^®^ Corporation, St. Paul, MN, USA), it is shown that it increased the chloride threshold (CR) by 54% and reduced the corrosion velocity (*i_corr_*) by 46% when compared to the reference concrete mix in the report.

## 3. Results and Discussion

### 3.1. Performance in Terms of Service Life

The concrete performance was assessed in terms of service life, with respect to steel reinforcement corrosion, with and without the presence of the bioproduct and AACI corrosion inhibitor in two different microenvironments: XS2 and XS3 (Table 2). The calculus variables used for the probabilistic analysis of RC service life are presented in Table 3. The initiation and propagation periods resulting from chloride action are defined by EN 206-1 [43] following the recommendations of fib Bulletin 34 (2006).

**Figure 3:**
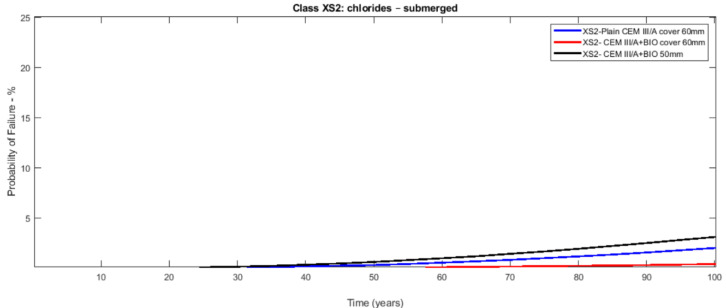
Probabilistic service life results of CEM III/A concrete—exposure class XS2 (submerged)—without and with bioproduct for a cover of 60 and 50 mm.

**Figure 4:**
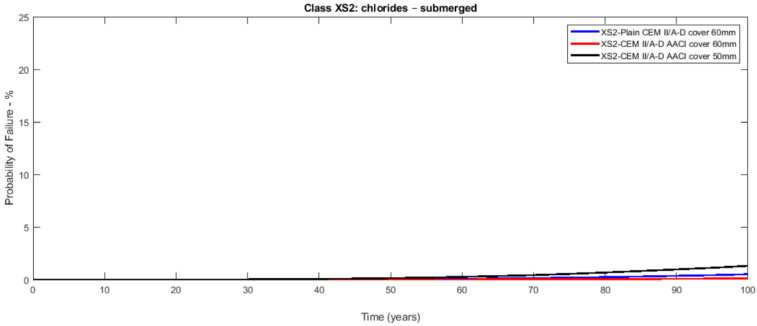
Probabilistic service life results of CEM II/A-D concrete—exposure class XS2 (submerged)—without and with AACI for a cover of 60 and 50 mm.

**Figure 5:**
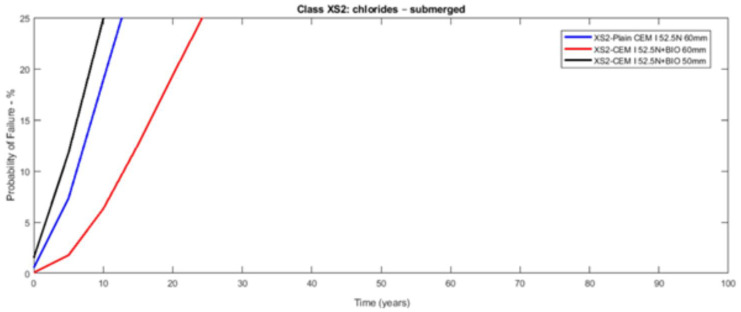
Probabilistic service life results of CEM I 52.5N concrete—exposure class XS2 (submerged)—without and with bioproduct for a cover of 60 and 50 mm.

**Figure 6:**
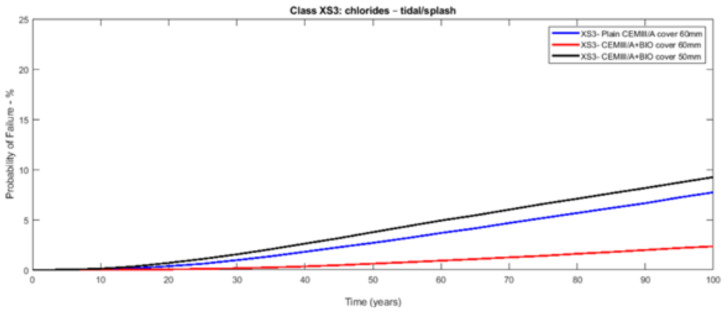
Probabilistic service life results of CEM III/A concrete—exposure class XS3 (tidal/splash)—without and with bioproduct for a cover of 60 and 50 mm.

**Figure 7:**
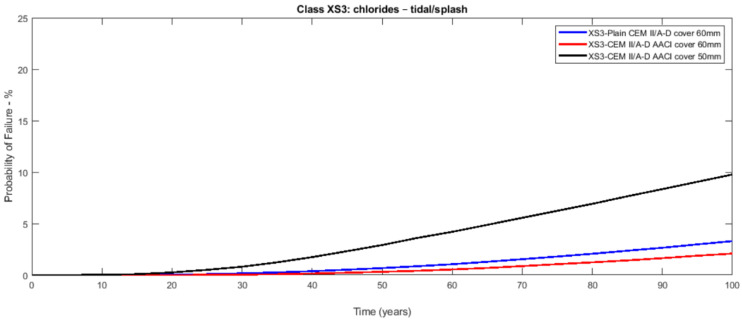
Probabilistic service life results of CEM II/A-D concrete—exposure class XS2 (submerged)—without and with AACI for a cover of 60 and 50 mm.

**Figure 8:**
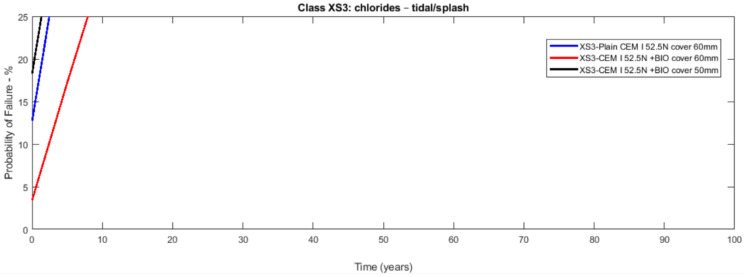
Probabilistic Service life results of CEM I 52.5N concrete—exposure class XS3 (tidal/splash)—without and with bioproduct for a cover of 60 and 50 mm.

The probabilistic study of the distribution of service life was conducted utilising the statistical parameters of the involved variables: Table 3 presents the mean values along with the standard deviation with their distribution laws. The estimated design service life is represented through the likelihood of failure *P_f_* as a time function [17,18]. Figure 3, Figure 4, Figure 5, Figure 6, Figure 7 and Figure 8 show that there are major differences for each concrete composition if the influence of corrosion inhibitors is considered. Considering the same level of probability of failure, *P_f_*, compared to concrete mixes without inhibitors, there is an increase in the design service life values for concrete compositions with bioproduct and AACI.

The previous results show that the bioproduct provides a significant contribution to the increase of the service life of RC structures with CEM III/A. CEM III/A and CEM II/A-D concretes are commonly used for structures exposed to maritime environments such as bridges. CEM I concrete (Figure 5 and Figure 8) is presented here for comparison as a plain mixture without the additives of the previous two. Although CEM II/A-D had a very good performance in both XS2 and XS3 environments (Figure 4 and Figure 7), the comparison of concrete CEMIII/A+BIO and concrete CEM II/A-D+AACI (Figure 6 and Figure 7) shows the benefit of the use of bioproduct (BIO) in the corrosion inhibition context. The addition of the bioproduct lowers the probability of failure of the CEM III/A mix from 7.5 to 2.5% (67% reduction), and more importantly, the repair solution with self-healing behaviour increases the RC service life and enables a decrease in the cover thickness from 60 mm to 50 mm in XS2 environment (Figure 6), while maintaining almost exact performance. This outstanding improvement is explained by the influence of the bioproduct on the properties of the concrete that are directly related to the corrosion initiation and propagation periods. For the same exposure class and concrete cover, the benefits of using CEM III/A+BIO in comparison to plain concrete (Figure 6) are greater than the benefit of CEM II/A-D+AACI in comparison to plain concrete in XS3 environments (Figure 7). However, in the case of the XS2 environment, CEM II/A-D and CEM III/A+BIO have similar performances (Figure 3 and Figure 4). Considering chloride exposure classes XS2 and XS3, the service life results for compositions with bioproduct had better or similar performance when compared to plain concrete mixes and mixes with AACI. The results in Figure 5 and Figure 8 show performance differences, as it is evident that CEM I 52.5 N is not tailored for structure repair in a chloride environment, and the bioproduct does not enhance the concrete service life to suitable levels. The CEM I concrete was chosen to emphasise the suitability of different types of cement for various application conditions and to show that the developed bioproduct solutions are tailored for specific substrates, such as CEMIII/A. The improvement in the performance of the compositions where corrosion inhibitors are modelled is clear when these are compared with plain concrete performance, which is consistent with the experimental results of the inhibitors analysed (Table 3). The results also showed that there was a 30% decrease of the chloride migration coefficients when the bioproduct was used, in comparison to plain compositions, which is in agreement with [31,32].

### 3.2. Impact on the Concrete Embodied Carbon

The impact of the use of a bioproduct or other type of corrosion inhibitor does not relate only to the service life of a structure. The use of resources and the environment needs to be taken into consideration as well. On top of ensuring a service life of 50 or 100 years, structures also need to use larger quantities of systems and materials. Choosing an appropriate composition of concrete increases the LCA impact and relevance when it is correlated to the durability of the structure. Here, the efficacy of a low carbon project should therefore be considered by its expected service life along with the energy expenditure and embodied carbon [47,48].

Table 4 presents the embodied carbon associated with the production of concrete CEM I, CEM II/A-D, and CEM III/A.

The results show that the bioproduct creates more opportunities to use low-carbon concretes with improved service life. The increase of RC service life via self-healing techniques delays the need for CO_2_ associated with heavy replacement infrastructure. The average embodied CO_2_e effect of concrete in the UK is approximately 100 kg CO_2_e/tonne. The previous modelling results show that the bioproduct leads to a significant contribution to the increase of RC service life with CEM III/A and cover reduction. Even if the bioproduct leads to a reduction in the concrete cover of 10 mm (from 60 to 50 mm), with approximately 5% less RC section, this means that there is an embodied carbon reduction equal to 472 kg CO_2_e/m^3^ of OPC concrete × 0.05 = 23.6 kg CO_2_e/m^3^ reduction (9.4 kg CO_2_e/tonne). Adding the CO_2_ savings from the use of CEM III/A instead of OPC means a total saving of approximately 20 kg CO_2_e/tonne of RC. The data within Table 4 are based on UK information regarding the indicative embodied CO_2_ (ECO_2_) figures for water, CEM I, GGBS, silica fume, sand gravel from Bath, and SIMAPRO inventories of carbon and energy [47]; these are also “cradle-to-gate” values [48,49,50].

The previous results show the environmental benefit of CEM III/A in comparison to CEM II/A-D, more specifically an embodied carbon reduction of more than 20% in comparison to CEM II and half of the CEM I, corresponding to a decrease of 250 kg of CO_2_e emissions per m^3^ of concrete.

## 4. Conclusions

A study was implemented to analyse the benefit of an iron-respiring bioproduct used to create self-healing behaviour in concrete in order to minimise corrosion in structures in maritime environments. Three different concretes, CEM I 52.5N, CEM II/A-D, and CEM III/A were tested with and without an iron-respiring bioproduct (BIO) and an added admixture corrosion inhibitor (AACI). The results show that the bioproduct had a significant contribution to the increase of the service life of RC structures with CEM III/A.

Concrete CEM III/A had 30% less embodied carbon than concrete CEM II/A-D. It is shown that the use of bioproduct in CEM III/A concrete enabled a probability of steel reinforcement corrosion lower than 2.3% for 100 years of service life, with a significant impact on the environment. The repair solution with self-healing behaviour increased RC service life and enabled the decrease of the cover thickness from 60 mm to 50 mm in an XS2 environment. In both the XS2 and XS3 environments, the comparison of CEM III/A+BIO and CEM II/A-D+AACI showed the benefit of the use of the bioproduct in the corrosion inhibition context, besides contributing to an embodied carbon reduction of more than 20%.

The presence of cracks leads to a lack of integrity of RC structures, compromising their safety, serviceability, and durability. At the first stage in maritime environments, the migration of chlorides is a major cause for that, and the development of flexible solutions able to mimic nature is essential to increase the initiation and propagation periods of RC corrosion. Self-healing concrete provides RC structures and infrastructures with the ability to adapt to different environments. In this paper, a bacteria-based solution is explored in the chloride environment context. Its potential for the improvement of a structure’s resilience is emphasised. It is demonstrated that the potential of self-healing concrete to improve RC structures’ service life is combined with a reduction of the use of resources such as energy and, ultimately, with significant benefits for the environment.

## Figures and Tables

**Table 1:** Concrete compositions tested and test results at the age of 28 days.

Concrete Composition	Binder Constituents	Dosage kg/m^3^	W/c Ratio	*f_cm_*^(1)^ MPa	*D*_0_^(2)^ m^2^/s	*i_corr_*^(3)^ μA/cm^2^
CEM I 52.5N	>95% clinker	450	0.45	46	16.7 × 10^−12^	1.0
CEM II/A-D	8% SF; >95% clinker	320	0.48	41	2.0 × 10^−12^	1.0
CEM III/A	60% GGBS; 40% clinker	450	0.45	47	5.5 × 10^−12^	0.20
CEM I 52.5N + Bio	>95% clinker	450	0.45	49	12.3 × 10^−12^	0.95
CEM III/A + Bio	60%GGBS; 40% clinker	450	0.45	44	3.9 × 10^−12^	0.19

SF—silica fume; FA—fly ash: GGBS—ground granulated blast furnace slag, ^(1)^ Mean cubic compressive strength. Moulds with 150 mm edge tested following EN 12390-1 (2009), ^(2)^ Migration coefficient using rapid non-steady state chloride test (NT Build 492), ^(3)^ Corrosion current density using electrical resistivity tests (AASHTOTP 95).

**Table 2:** Environmental exposure classes of corrosion: EN 1992-1-1 (2004) [46] and EN 206-1 (2005) [43].

Schematic view of environmental exposure environments 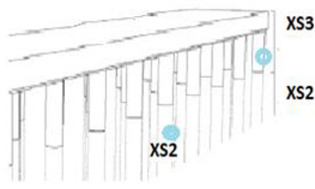	Type of environment	Classes of European Equivalent	Environmental exposure classes description
	Chlorides	XS2	Submerged areas that are in direct contact with seawater.
		XS3	Spray, tidal, and splash zones.

**Table 3:** Chlorides class and XS2 (submerged) and XS3 (tidal/splash)—calculus variables.

	Concrete					
Variables	Plain CEM I 52.5N	Plain CEM II/A-D	Plain CEM III/A	CEM I 52.5N + Bio	CEM II/A-D + Corrosion Inhibitor (AACI) [17]	CEM III/A + Bio
Cover, *c* (*c_nom_*)	60 mm	60 mm	60 mm	60 mm	60 mm	60 mm
Testing age, *t*_0_	28 days	28 days	28 days	28 days	28 days	28 days
Chloride threshold, *C_R_*	0.3% XS2/0.4% XS3 (binder wt)	0.3% XS2/0.4% XS3 (binder wt)	0.3% XS2/0.4% XS3 (binder wt)	0.3% XS2/0.4% XS3 (binder wt)	0.46% (binder wt)	0.3% XS2/0.4% XS3 (binder wt)
Chloride surface amount, *C_s_*	3.4% (binder wt)	4.1% (binder wt)	3.4% (binder wt)	3.4% (binder wt)	4.1% (binder wt)	3.4% (binder wt)
Temperature parameter *K_D,T_*	0.8	0.8	0.8	0.8	0.8	0.8
Rel. humidity parameter *K_D,RH_*	1	1	1	1	1	1
Curing parameter *K_D,C_*	0.75 (XS2); 2.4 (XS3)	0.75 (XS2); 2.4 (XS3)	0.75(XS2); 2.4 (XS3)	0.75 (XS2); 2.4 (XS3)	0.75 (XS2); 2.4 (XS3)	0.75 (XS2); 2.4 (XS3)
Corrosion current density, *i_corr_*	1.0 μA/cm^2^	1.0 μA/cm^2^	0.20 μA/cm^2^	0.95 μA/cm^2^	0.17 μA/cm^2^	0.19 μA/cm^2^
Tensile strength, *f_td_*	3 MPa	3 MPa	3 MPa	3 MPa	3 MPa	3 MPa
Steel bar diameter, *φ*_0_	8 mm	8 mm	8 mm	8 mm	8 mm	8 mm

*i_corr_ (CEM II/A-D AACI) = (1–0.83) i_corr_ (plain) = 0.17 icorr (plain), i_corr_(CEM III) = (1–0.8) i_corr_ (CEM I plain) = 0.2 icorr (CEM I plain), i_corr_(CEM I+BIO) = (1–0.05) i_corr_ (CEM I plain) = 0.95 icorr (CEM I plain), i_corr_(CEM III+BIO) = (1–0.81) i_corr_ (CEM I plain) = 0.19 icorr (CEM I plain).*

**Table 4:** Embodied carbon associated with the production of concrete CEM I, CEM II/A-D, and CEM III/A.

Cement (Binder)	Compositions	Embodied Carbon—(kg CO_2_e/kg)	Dosage	w/c	Embodied Carbon—(kg CO_2_e/m^3^) Concrete
Type			(kg/m^3^)		
CEM I (OPC)	>95% clinker	0.95	450	0.45	472
CEM II/A-D	8% SF; >95% clinker	0.88	320	0.48	325
CEM III/A	40% clinker; 60% GGBS	0.39	450	0.45	220

## Data Availability

The data presented in this study are available in cited references and on request from the corresponding author.
